# Pleomorphic adenoma of the parotid gland presenting as extensively ossified lesion with bone infiltration: a case report

**DOI:** 10.1016/j.bjorl.2021.07.014

**Published:** 2021-11-04

**Authors:** Marcel Mayer, Ruben Thoelken, Monika Jering, Bruno Märkl, Abbas Agaimy, Johannes Zenk

**Affiliations:** a Department of Otolaryngology, Head and Neck Surgery, University Hospital Augsburg, Germany; b Institute of Pathology and Molecular Diagnostics, University Hospital Augsburg, Germany; c Institute of Pathology, Friedrich-Alexander-University Erlangen-Nürnberg, University Hospital Erlangen, Germany

## Introduction

Salivary gland tumors account for 6% of all head and neck tumors. Approximately 80% of these tumors occur in the parotid gland. About 80% of parotid gland tumors are benign. Pleomorphic Adenoma (PA) is the most common salivary gland tumor overall (50%–70%).^1^ Although PA is a benign tumor, it can in rare cases result in benign metastases or malignant transformation^2^ and therefore is treated surgically. In some cases, surgical removal can be extremely challenging due to unusual localization or extensive growth. The histological patterns of a PA can either be myxoid (>80% of stromal component), cellular (20%–30% of stromal component), or classical (30%–50% of stromal component).^3^ Rare cases of extensive metaplastic bone formation in PA have been reported,^4^ but osteodestructive growth in a PA of the parotid gland has not been described before. Herein, we present a case of a patient with a markedly ossified PA of the parotid gland, which showed osteodestructive growth.

## Case report

**Figure 1:**
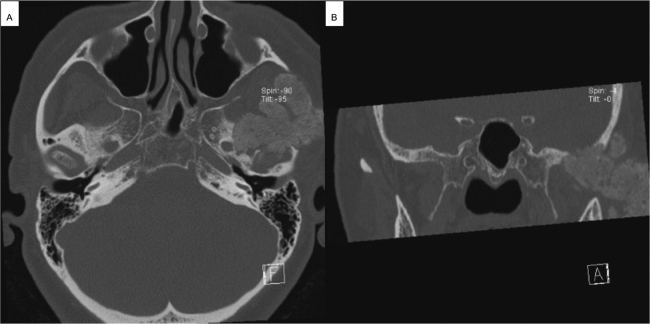
(A) Axial CT scan showing the osseous tumor of the left parotid gland reaching the foramen ovale of the skull base. (B) Coronal CT scan showing the tumor of the left parotid gland with osteodestruction of the left middle cranial fossa.

**Figure 2:**
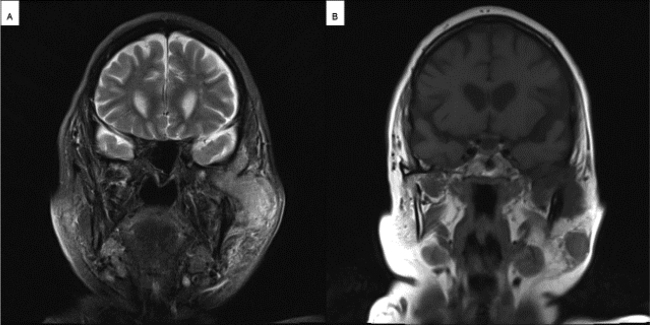
(A) Coronal T2 weighted MRI scan showing extensive temporomandibular growth of the left parotid tumor. (B) Coronal T1 weighted MRI scan showing the primary parotid tumor invading the temporomandibular joint and a synchronous tumor of the parotid tail.

An 80-year-old female was admitted to the Department of Otorhinolaryngology of the University Hospital of Augsburg with a history of swelling of the left cheek that first occurred three months ago. There was a poorly demarcated hard mass in the left parotid region with intact facial nerve function on clinical examination. On ultrasound examination, the mass showed a homogenous pattern. The mass had sharp borders and poor vascularity on the color-coded doppler sonography. The deeper part of the tumor was inaccessible to sonographic evaluation. CT (Fig. 1) and MRI (Fig. 2) imaging showed a huge tumor involving both the superficial and the deep lobe of the parotid gland with extension into the infratemporal fossa. There was a remarkable osteodestruction in the middle cranial fossa, involving the temporal bone and, to less extent, the sphenoidal bone with a bony defect of about 2.4 cm diameter. The temporomandibular joint showed signs of destruction. The tumor was lobulated with a clear margin and calcified deposits. In the superficial parotid tail, there was a second tumor (Fig. 2).

Core needle biopsy showed asclerosing epithelial neoplasia with glandular structure and preserved myoepithelial layers. The immunohistochemical staining confirmed a biphasic differentiation with nuclear positivity for p63 in the basal cells and luminal positivity for CK7. DOG1 was negative. The pathology report concluded pleomorphic adenoma without significant signs of malignancy. Therefore, the decision was made for surgical resection of the lesion.

**Figure 3:**
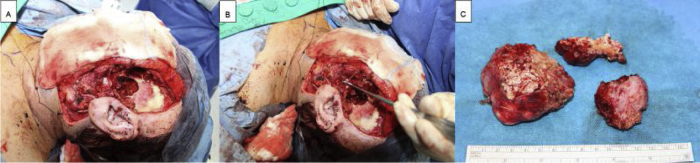
(A) Intraoperative image after surgical removal of the parotid tumor. (B) Intraoperative image showing a branch of the facial nerve. (C) Intraoperative image showing the osseous tumor after surgical removal.

**Figure 4:**
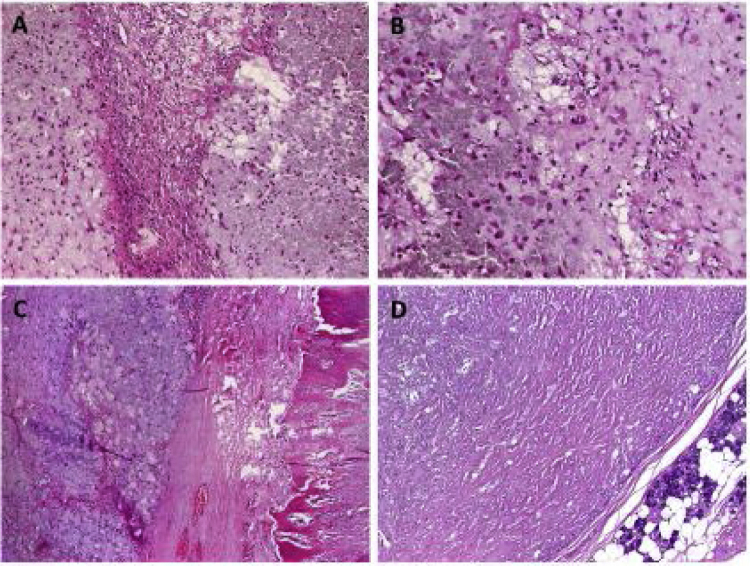
(A) Overview of the pleomoprhic adenoma showing chondromyxoid tissue (left) juxtaposed to oxalat-like stromal deposits. (B) Higher magnification of the transitional zone between the two components. (C) Some tumor lobules showed extensive metaplastic ossification. (D) The second pleomorphic adenoma showed extensive stromal sclerosis but no other features.

The modified Blair incision was extended cranially to the temporal area and caudally to the neck. A dissection of the temporal muscle, sternocleidomastoid muscle, and parotid capsule, as well as the tumor, was performed. Superficial parotidectomy was performed. Bony edges were smoothed using a diamond drill. Temporalis muscle was used for filling the defect. Redon drain was placed, and wound closure was performed (Fig. 3). Postoperatively, the patient showed facial palsy (House Brackmann Score IV). On follow up two weeks after surgery, facial palsy was unchanged. On histopathological examination, the lesion of the superficial parotid tail was shown to be PA. More interestingly, the ossified cranial parotid lesion was shown to be a synchronous PA with excessive proportion of calcium pyrophosphate leading to marked ossification (Fig. 4). There were no signs of malignancy. Complete resection was confirmed histpathologically. The patient was reccurence-free 26 months after operation.

## Discussion

Herein, we present the rare case of a female patient with an extensively ossified Pleomorphic Adenoma (PA) of the parotid gland, which showed infiltration of the zygomatic and the mandibular bone.

Stennert et al. examined 100 cases of PA. Focal capsule absence was found in 43% of the cases. This finding was more prominent in myxoid tumors and involved 28% of the entire circumference. A thin capsule was defined as an area with a thickness of less than 20 micrometers. Pseudopodia (40% of cases) was defined as a tumor island separated from the main tumor but within the capsule. Further, satellite nodules (16% of cases) were defined as separated from both the capsule and the main tumor.^5^ Depending on the stromal component of the tumor, PA can either be myxoid (>80% of stromal component), cellular (20%–30% of stromal component), or classical (30%–50% of stromal component). The stroma can range from myxoid, chondroid, lipomatous to osseous.^3^ Extensive bone formation in PA has been described in sporadic cases,^4^ but bone infiltration by a benign, extensively ossified PA of the parotid gland has, to the best of our knowledge, not been described before in the literature. A possible explanation for osteodestruction in the present case may be extensive sclerosis of the tumor causing mechanical pressure and subsequently destruction of the adjacent bones.

Given the bone destruction of the cranial fossa and the proximity to the brain neurosurgical/neurological support in surgical planning – and if necessary, during surgery itself – should be considered in cases like these. In this case there was no infiltration of the dura, so the tumor was resected, and the defect was closed without neurosurgical involvement.

On ultrasound examination, PA presents as lobulated lesion with well-defined margins. The tumor is usually homogenous with low vascularity. In the present case, ultrasonographic appearance was in accordance with these features.

MRI is the second choice for preoperative diagnose, especially in extensive tumor growth. On MRI, PA shows low to intermediate intensity on T1 weighted image, which was also found in the present case. CT is rarely needed but can be useful in showing boney involvement. A well-defined, sometimes lobulated mass, with either heterogeneous or homogeneous contrast enhancement can be demonstrated. In the present case, the CT scan displayed an extensive bony defect of the middle cranial fossa caused by the hyperdense lesion. Knowing the final histopathology, an extensively ossified PA would have been a likely differential diagnosis in the synopsis of the ultrasonographic and the cross-sectional scanning findings.

## Conclusion

The present case displays a patient with an ossified pleomorphic adenoma of the left parotid gland, which showed osteodestruction of the zygomatic and mandibular bone and extensive growth in the middle cranial fossa. It emphasizes the occurrence of pleomorphic adenomas with extensive bone formation which is only rarely described in the current literature. Further it shows that PA of the parotid gland, although being a benign tumor, can show locally destructive growth. Eventually, PA cannot be excluded as a differential diagnosis in case of an ossified lesion of the parotid gland with local osteodestruction.

## Conflicts of interest

The authors declare no conflicts of interest.
